# Neostigmine Attenuates Proinflammatory Cytokine Expression in Preoptic Area but Not Choroid Plexus during Lipopolysaccharide-Induced Systemic Inflammation

**DOI:** 10.1155/2018/9150207

**Published:** 2018-10-09

**Authors:** Andrzej P. Herman, Dorota Tomaszewska-Zaremba, Marta Kowalewska, Aleksandra Szczepkowska, Małgorzata Oleszkiewicz, Agata Krawczyńska, Maciej Wójcik, Hanna Antushevich, Janina Skipor

**Affiliations:** ^1^The Kielanowski Institute of Animal Physiology and Nutrition, Polish Academy of Sciences, 05-110 Jabłonna, Poland; ^2^Institute of Animal Reproduction and Food Research, Polish Academy of Sciences, 10-748 Olsztyn, Poland

## Abstract

The study was designed to examine whether the administration of neostigmine (0.5 mg/animal), a peripheral inhibitor of acetylcholinesterase (AChE), during an immune/inflammatory challenge provoked by intravenous injection of bacterial endotoxin—lipopolysaccharide (LPS; 400 ng/kg)—attenuates the synthesis of proinflammatory cytokines in the ovine preoptic area (POA), the hypothalamic structure playing an essential role in the control of the reproduction process, and in the choroid plexus (CP), a multifunctional organ sited at the interface between the blood and cerebrospinal fluid in the ewe. Neostigmine suppressed (*p* < 0.05) LPS-stimulated synthesis of cytokines such as interleukin- (IL-) 1*β*, IL-6, and tumor necrosis factor (TNF) *α* in the POA, and this effect was similar to that induced by the treatment with systemic AChE inhibitor—donepezil (2.5 mg/animal). On the other hand, both AChE inhibitors did not influence the gene expression of these cytokines and their corresponding receptors in the CP. It was found that this structure seems to not express the neuronal acetylcholine (ACh) receptor subunit alpha-7, required for anti-inflammatory action of ACh. The mechanism of action involves inhibition of the proinflammatory cytokine synthesis on the periphery as well as inhibition of their de novo synthesis rather in brain microvessels and not in the CP. In conclusion, it is suggested that the AChE inhibitors incapable of reaching brain parenchyma might be used in the treatment of neuroinflammatory processes induced by peripheral inflammation.

## 1. Introduction

It is well known that peripheral inflammation caused by bacterial and viral infections and inflammatory diseases may differently affect the central nervous system (CNS) and its constituents including the hypothalamic-pituitary-gonadal (HPG) axis responsible for reproduction [1, 2]. The most common bacterial endotoxin used as a model of immune stress without necessity of infecting the animal with active pathogen in the experiments evaluating the impact of an immune challenge on reproductive functions is lipopolysaccharide (LPS). LPS is a well-characterized pathogen-associated molecular pattern found in the outer leaflet of the outer membrane of Gram-negative bacteria. It consists of lipid A molecule (endotoxin), core sugars, and O-antigen [3]. After its release, endotoxin is bound by circulating LPS-binding protein (LBP) and then transferred to cluster of differentiation (CD) 14, which can be found either in a soluble form or can be found linked to the cell surface. CD14 splits LPS aggregates into monomeric molecules and presents them to the toll-like receptor (TLR) 4–myeloid differentiation-2 (MD-2) complex located in the cell membrane. When the lipid A is recognized by the TLR4-MD-2 complex, it triggers the innate immune signaling pathway, thereby leading to the activation of transcription factor NF*κ*B and production of chemokines and cytokines, adhesive and costimulatory molecules, and enzymes [4, 5]. In our previous study, it was demonstrated that constitutive mRNA expression of TLR4 is present in the preoptic area (POA), anterior and medial basal hypothalamus. It is important since in these parts, gonadotropin-releasing hormone (GnRH) neurons (a key player in regulation of the HPG axis) are localized [2]. In ewes, peripheral administration of LPS results in significant elevation of interleukin- (IL-) 1*β*, tumor necrosis factor (TNF) *α*, and IL-6 in the hypothalamus [6–8]. These proinflammatory cytokines influence the neuroendocrine system–regulating activity of GnRH neurons by acting through corresponding receptors widely expressed in the region of the hypothalamus [6, 8–10].

Among endogenous mechanisms involved in the regulation of immune response and cytokine secretion is the cholinergic anti-inflammatory pathway. This mechanism could be activated by stimulation of the vagus nerve thereby increasing the acetylcholine (ACh) secretion [11]. It was previously found that ACh acting through activation of neuronal acetylcholine receptor subunit alpha-7 (CHRNA7) reduces LPS-stimulated secretion of IL-1*β*, IL-6, and TNF*α* [12]. Such anti-inflammatory mechanism could be also activated by pharmacological inhibition of the acetylcholinesterase (AChE) activity. It is worth mentioning that CHRNA7 is widely expressed in the brain, and the increasing number of evidences suggests that this receptor plays an important role in protecting the brain against neurodegenerative and neuroinflammatory processes [13]. In our previous study on the sheep, it was shown that peripheral administration of the systemic AChE inhibitor, rivastigmine, reduced inflammatory-induced synthesis of IL-1*β* in the hypothalamus [7], and this treatment was sufficient to diminish the inhibitory effect of LPS-induced inflammation on the secretion of GnRH and luteinizing hormone (LH) [14]. In another study on mice, it was suggested that also peripheral-acting AChE inhibitors, unable to cross brain barriers, may influence the transition of the inflammatory signal to the CNS [15]. Indeed, just recently, we have demonstrated that administration of neostigmine, the AChE inhibitor unable to reach the brain parenchyma, may successfully reverse some negative effects of inflammation on the GnRH neurons [16]. This fact suggests the attenuation of the neuroinflammatory processes in the region of the hypothalamus. It is not known, however, if neostigmine affects the LPS receptor expression and its signaling in the hypothalamus.

It has been established that the lipid A colocalizes with CD14, TLR4, and NF*κ*B (translocated into the nucleus) in the choroid plexus (CP), a multifunctional organ situated at the interface between the blood and cerebrospinal fluid (CSF) [17]. In our recent studies, it was shown that activation of the LPS receptor in the CP induces mRNA expression of TLR4, CD14, and proinflammatory cytokines and their receptors and then induces cytokine secretion into the cerebrospinal fluid (CSF) [18–20]. Unfortunately, information about neostigmine effect on CP response to LPS challenge is lacking. It would be interesting in view of that fact that CP is innervated by cholinergic nerves, with endings associated with blood vessels and epithelial cells [21]. Choline acetyltransferase and neuronal acetylcholine receptor subunits are expressed at different levels across CP ventricular sites [22]; however, detailed information concerning receptor subunit localization is missing. In CP, the vascular endothelium is separated from the epithelium by a stromal matrix that contains numerous immunocompetent cells in which CHRNA7 is widely expressed. Moreover, the cellular composition of the stroma can be dynamically altered during inflammation through recruitment of circulating immune cells, such as lymphocytes, neutrophils, and monocytes [23].

Therefore, the present study was designed to determine the effect of the peripherally acting AChE inhibitor—neostigmine—on the mRNA expression of proinflammatory cytokines and their corresponding receptors in the hypothalamus and CP and proinflammatory cytokine synthesis during acute inflammation induced by LPS injection in ewes.

## 2. Materials and Methods

### 2.1. Animals

The study was carried out on adult, 2-year-old Blackhead ewes during the reproductive season (September–October). The ewes were maintained in good conditions, i.e., their body condition was estimated at 3 in a five-point scale [24]. The animals were acclimated to the experimental conditions for one month. The ewes had constant visual contact with each other as to avoid isolation stress. The animals were fed a constant diet consisting of commercial concentrates and had ad libitum access to hay and water, according to the recommendations of the National Research Institute of Animal Production for adult ewes [25]. In order to standardize experimental conditions, the stages of the ewe estrous cycle were synchronized by the Chronogest® CR (Merck Animal Health, Boxmeer, The Netherlands) method using an intravaginal sponge impregnated with 20 mg of a synthetic progesterone-like hormone. All ewes had Chronogest® CR sponges placed for 14 days. Following sponge removal, the ewes received an intramuscular injection of 500 IU pregnant mare's serum gonadotropin (PMSG) (Merck Animal Health, Boxmeer, The Netherlands). The experimental procedure was performed 24 h after PMSG injection. In treated animals, the immune stress was induced by the intravenous (iv.) injection of LPS from *Escherichia coli* 055:B5 (Sigma-Aldrich, St. Louis, MO, USA) in a dose of 400 ng/kg, dissolved in saline (0.9% *w*/*v* NaCl) (Baxter, Deerfield, IL, USA) at a concentration of 10 mg/l.

All procedures were performed with agreement of the Local Ethics Committee of Warsaw University of Life Sciences–SGGW (Warsaw, Poland; authorization no. 50/2013; date of approval: September 18, 2013).

### 2.2. Experimental Procedures

Venous catheters were implanted into the jugular vein on the day prior to the experiment. The ewes (*n* = 36) were randomly divided into six experimental groups (Table 1). Half hour prior to LPS/saline treatment, the animals were slowly intravenously treated with saline (groups 1 and 2) or suitable AChE inhibitor: donepezil (systemic) (groups 3 and 5) or neostigmine (peripheral) (groups 4 and 6). Just before euthanasia, the concentration of IL-1*β* was measured in the jugular blood samples (6 ml). The animals were euthanized 3 h after LPS or saline administration, and the brains were rapidly removed from the skulls. From the ovine brains, the CP and the POA—the hypothalamic structure where the great majority of GnRH neurons have their bodies located—were dissected [26]. The structures were dissected according to the stereotaxic atlas of the sheep brain [27]. Landmarks were mammillary body, median eminence, and optic chiasm. The depth of the cut was 2.5 to 3 mm for the POA. All tissues after collection were frozen immediately in liquid nitrogen and then stored at −80°C.

### 2.3. Assays

#### 2.3.1. Determining the Relative Gene Expression

Total RNA from the CP and POA was isolated using the components of NucleoSpin® RNA Kit (MACHEREY-NAGEL Gmbh & Co.; Düren, Germany) according to a manufacturer's instruction. The purity and concentration of isolated RNA were spectrophotometrically quantified by measuring the optical density at 230, 260, and 280 nm in a NanoDrop 1000 instrument (Thermo Fisher Scientific Inc., Waltham, USA). The RNA integrity was verified by electrophoresis using 1% agarose gel stained with ethidium bromide (Sigma-Aldrich, St. Louis, MO, USA). A Maxima™ First Strand cDNA Synthesis Kit for RT-qPCR (Thermo Fisher Scientific Inc., Waltham, USA) was used to prepare cDNA synthesis. As a starting material for this PCR synthesis, 2 *μ*g of total RNA was used.

Real-time RT-PCR was carried out using HOT FIREPol EvaGreen® qPCR Mix Plus (Solis BioDyne, Tartu, Estonia) components and HPLC-grade oligonucleotide primers synthesized by Genomed (Warsaw, Poland). Specific primers for determining the expression of housekeeping genes and genes of interest were chosen based on our previous studies (Table 2). One tube contained 4 *μ*l PCR Master Mix (5x), 14 *μ*l RNase-free water, 1 *μ*l primers (0.5 *μ*l each, working concentration 0.25 *μ*M), and 1 *μ*l cDNA template. The tubes were run on a Rotor-Gene 6000 (Qiagen, Duesseldorf, Germany). The following protocol of PCR reaction was used: 95°C in 15 min for activating Hot Start DNA polymerase and finally the PCR including 30 cycles at 95°C in 10 sec for denaturation, 60°C in 20 sec for annealing, and 72°C in 10 sec for extension. After the cycles, a final melting curve analysis under continuous fluorescence measurements was performed to confirm the specificity of the amplification.

Relative gene expression was calculated using the comparative quantification option [30] of the Rotor-Gene 6000 software version 1.7 (Qiagen, Dusseldorf, Germany). Three housekeeping genes, glyceraldehyde-3-phosphate dehydrogenase (GAPDH), *β*-actin (ACTB), and histone deacetylase 1 (HDAC1), were examined. The mean expression of these housekeeping genes was used to normalise the expression of the analysed genes. The results are presented in arbitrary units, as the ratio of the target gene expression to the mean expression of the housekeeping genes.

#### 2.3.2. ELISA Assay for IL-1*β* Concentration in the Blood

The concentrations of IL-1*β* in the blood serum were determined using a commercial IL-1*β* ELISA kit (cat no. ESH0012; Wuhan Fine Biotech Co. Ltd., Wuhan, China). The blood samples were kept overnight at 4°C and then centrifuged for 20 min at 1000 ×g at 4°C. The supernatants were aliquoted and stored until assay at −80°C. The assay was performed according to the manufacturer's instructions. The incubation of plates and absorbance measurement at 450 nm was performed using a VersaMax reader (Molecular Devices LLC., Sunnyvale, California, USA). The sensitivity of the assay was 18.75 pg/ml.

#### 2.3.3. ELISA Assay for IL-1*β*, IL-6, and TNF*α* Concentrations in the Hypothalamus

The concentrations of IL-1*β*, IL-6, and TNF*α* in the POA were determined using commercial IL-1*β*, IL-6, and TNF*α* ELISA kits (Cusabio Biotech Co. Ltd., Wuhan, China) designed and validated for the sheep. The hypothalamic tissues were homogenized in 1 ml of cold phosphate buffered saline (0.02 M), and then homogenates were subjected to two freeze-thaw cycles to further break the cell membranes. Homogenates were then centrifuged for 5 min at 5000 ×g at 4°C. The supernatants were aliquoted and stored until assay at −80°C. Assays were performed according to the manufacturer's instructions. The incubation of plates and absorbance measurement at 450 nm was performed using the VersaMax reader (Molecular Devices LLC., Sunnyvale, California, USA). The sensitivities of assays were 3.9 pg/ml (IL-1*β*; cat no. CSB-E10115Sh), 2 pg/ml (IL-6; cat no. CSB-E10116Sh), and 3.12 pg/ml (TNF*α*; cat no. CSB-E13853Sh).

### 2.4. Statistical Analysis

The results were analysed using a two-way ANOVA. The examined factors were the inflammatory state and AChE inhibitor-treatment (donepezil or neostigmine). Before ANOVA was conducted, its two assumptions were checked: normality (Shapiro-Wilk's test) and homogeneity of the variances (Levene's test). When a significant treatment by time interaction was observed, a post hoc analysis was conducted to identify treatment effects. Fisher's least significant difference post hoc test was used to compare precompared values with posttreatment ones. Statistical significance was defined as *p* < 0.05.

The statistical analysis was performed using the STATISTICA 10 software (StatSoft Inc., Tulsa, OK, USA).

## 3. Results

### 3.1. Effect of AChE Inhibitors and LPS Administration on the Serum Concentration of IL-1*β*

**Figure 1:**
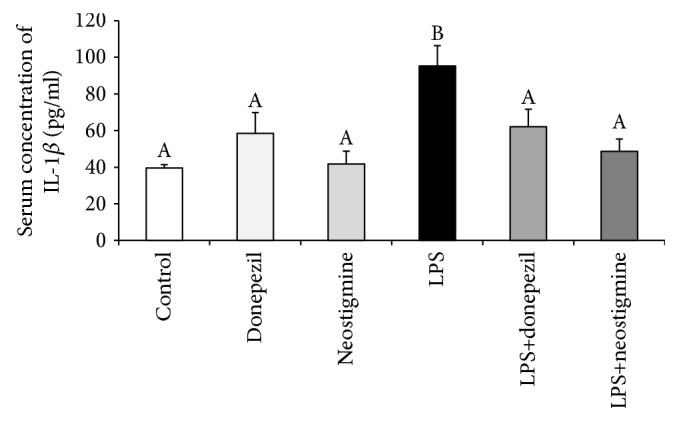
Effect of lipopolysaccharide (LPS; 400 ng/kg; iv.) and acetylcholinesterase inhibitors: donepezil (2.5 mg/animal; iv.) and neostigmine (0.5 mg/animal; iv.) injections on the serum concentration of IL-1*β* in the samples collected three hours after the LPS treatment. The data are presented as the mean value ± SEM. Different capital letters indicate significant differences according to a two-way ANOVA followed by Fisher's post hoc test. Statistical significance was defined at *p* < 0.05.

Injection of LPS increased (*p* < 0.05) concentration of IL-1*β* in the blood, whereas the intravenous administration of donepezil as well as neostigmine successfully abolished LPS-induced increase (*p* < 0.05) in the serum concentration of this cytokine (Figure 1).

### 3.2. Effect of AChE Inhibitors and LPS Administration on the Levels of IL-1*β*, IL-6, and TNF*α* in the POA

**Figure 2:**
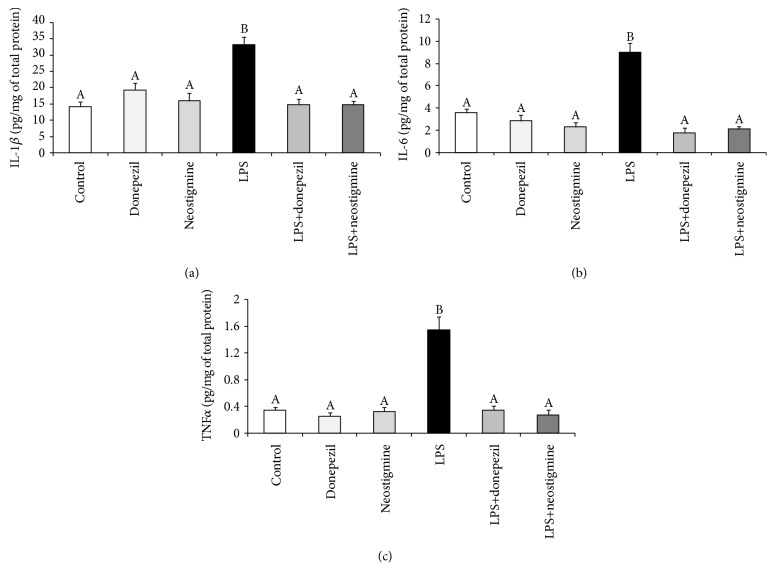
Effect of lipopolysaccharide (LPS; 400 ng/kg; iv.) and acetylcholinesterase inhibitors: donepezil (2.5 mg/animal; iv.) and neostigmine (0.5 mg/animal; iv.) injections on the level of proinflammatory cytokines—interleukin- (IL-) 1*β* (a), IL-6 (b), and tumor necrosis factor (TNF) *α* (c) in the preoptic area of the hypothalamus. The data are presented as the mean value ± SEM. Different capital letters indicate significant differences according to a two-way ANOVA followed by Fisher's post hoc test. Statistical significance was defined at *p* < 0.05.

It was found that peripheral administration of bacterial endotoxin increased (*p* < 0.05) the content of such proinflammatory cytokines as IL-1*β*, IL-6, and TNF*α* in the POA. The intravenous treatment with donepezil as well as neostigmine prevented the LPS-induced increase (*p* < 0.05) in the level of all examined proinflammatory cytokines in the POA (Figure 2).

### 3.3. Effect of AChE Inhibitors and LPS Administration on the Gene Expressions of Proinflammatory Cytokines, Their Corresponding Receptors, and CHRNA7 in the POA

Endotoxin treatment stimulated (*p* < 0.05) mRNA expression of all examined proinflammatory cytokines and ILR1, IL1R2, IL-1RN, IL6ST, and TNFRSF1B in the POA. Moreover, endotoxin-induced inflammation increased (*p* < 0.05) the gene expression of CHRNA7 in this hypothalamic structure. On the other hand, administration of donepezil and neostigmine reduced (*p* < 0.05) stimulatory effect of LPS on the gene expression of proinflammatory cytokines but not on their corresponding receptor gene expression in the POA; however, the amount of transcript encoding IL-1*β* and IL-6 was still significantly higher (*p* < 0.05) in comparison to the control group. It is worth mentioning that the administration of AChE inhibitors did not influence the expression of all genes examined in animals not treated with endotoxin (Table 3).

### 3.4. Effect of AChE Inhibitors and LPS Administration on the Gene Expressions of Proinflammatory Cytokines, Their Corresponding Receptors, and CHRNA7 in the Ovine CP

In CP, LPS administration significantly (*p* < 0.05) increased mRNA expression of all investigated cytokines and their receptors, except ILR6. No effect of AChE inhibitors, donepezil and neostigmine, on gene expression of proinflammatory cytokines and their receptors was observed under both basal and LPS-challenged conditions (Table 4). Moreover, Real-Time PCR analysis showed that no mRNA encoding CHRNA7 is expressed in the ovine CP regardless of the immune status of animals.

## 4. Discussion

The present study demonstrated that peripheral AChE inhibitor, neostigmine, reduces the proinflammatory cytokine synthesis (including IL-1*β*, IL-6, and TNF*α* in the POA) during acute inflammation induced by bacterial endotoxin. The effectiveness of its action is similar to systemically acting AChE inhibitor—donepezil. These results strongly support the thesis that pharmacological activation of the cholinergic anti-inflammatory pathway on periphery is sufficient to reduce the transmission of the inflammatory signal to the CNS. Similar findings were reported in the studies on mice where peripheral AChE inhibitors effectively reduced the LPS-dependent increase in the IL-1*β* synthesis in the hippocampus [15]. Neostigmine does not cross the blood–brain barrier (BBB) located in the endothelial cells of brain microvessels nor the blood-cerebrospinal barrier situated in the epithelial cells of the CP [31]. Therefore, decreased synthesis of central cytokines in the hypothalamus, especially in the case of animals treated with neostigmine, may result from the suppression of LPS-induced secretion of circulating proinflammatory cytokines, particularly IL-1*β*. In general, the source of IL-1*β* in the brain is differentiated. It may originate from (1) periphery and then is transported by brain barriers to reach the brain parenchyma or to enter the brain through the circumventricular organs [32–34], (2) de novo synthesis in the brain microvessel endothelium and CP [20, 35], and (3) local synthesis in microglial and dendritic cells, astrocytes, and even neurons [36]. Therefore, it may be expected that early after LPS administration, the inhibitory action of neostigmine on central IL-1*β* may result from reduction of IL-1*β* able to reach the brain and from its decreased de novo synthesis in the brain microvessels endothelium (BBB) as well as CP. It is worth mentioning that the BBB cells have been shown to produce not only IL-1*β* but also broad spectrum of pro- and anti-inflammatory cytokines. In the monkey, brain endothelial cells challenged *in vitro* with an immune stimulus, IL-1*β* or LPS, stimulated the release of IL-6, and the effect was greater in endothelia from aged animals [37]. Moreover, in the study on mouse brain endothelial cell culture, it was observed that these cells exhibit both constitutive and LPS-induced syntheses not only of IL-1*β* but also of IL-6, IL-10, TNF*α*, and granulocyte-macrophage colony-stimulating factor [38]. Also, the CP is considered to be a source of various pro- and anti-inflammatory cytokines [18, 39]. Having in mind the fact that cholinergic neurons have been found to directly contact the microvascular endothelium of BBB [40] and that CHRNA7 is expressed in the BBB cells [41], we suggest that attenuation of the inflammatory signal incoming from the periphery into the hypothalamus after neostigmine treatment results also from the reduced de novo synthesis of inflammatory mediators in the microvessel endothelial cells; however, this issue requires further detailed research. Despite the presence of immunocompetent cells in the CP stroma, the negligible expression of CHRNA7 and therefore no effect of neostigmine on de novo synthesis of proinflammatory cytokines were observed. In the CP, only increased mRNA expressions of IL-1*β*, IL-6, and TNF*α* as well as their receptors (except IL6R) following LPS administration were shown, which is generally consistent with previous studies suggesting that activated CP synthesizes a number of inflammatory mediators and expresses their corresponding receptors [18, 39]. It is worth mentioning that inflammatory cytokines are not accumulated in the cells, but they are continuously released during stimulation [42]. So, although the changes in the proinflammatory cytokine synthesis in the CP were assayed only at the level of transcription, it could be assumed that the changes in this cytokine production are generally parallel to changes in their gene transcription.

The results obtained in this study may confirm our previous observation that neostigmine treatment prevented inflammatory-dependent suppression of GnRH/LH secretion in ewes during the follicular phase of the estrous cycle [16]. The presented results provide a further evidence to prove the pivotal role of proinflammatory cytokines in the central mechanism disturbing the activity of the HPG axis (accompanying the inflammatory state). Although our study demonstrated that under inflammatory condition the expression of all examined proinflammatory cytokines was increased in the POA, the results of *in vivo* experiments indicate that central IL-1*β* and TNF*α* are the major proinflammatory cytokines mediating the LPS-induced suppression of GnRH and LH releases; the role of IL-6 seems to be insignificant [43–46]. Generally, the results of our previous studies and the present experiment strongly support the results of the study on mice [15] in which was suggested that the blood level of immune mediators in order to disturb the functioning of CNS has to enrich a critical level, and the reduction of the circulating level of proinflammatory cytokines under certain conditions may be sufficient for significant inhibition of LPS-induced synthesis of IL-1*β* in the CNS [15].

In summary, the study demonstrated that neostigmine, the peripheral inhibitor of AChE activity, may effectively suppress the synthesis of such central proinflammatory cytokines as IL-1*β*, IL-6, and TNF*α* in the region of the hypothalamus, essential for the central controlling of reproduction, stimulated by acute inflammation induced by intravenous injection of bacterial endotoxin. The mechanism of action involves inhibition of the proinflammatory cytokine synthesis on the periphery as well as inhibition of their de novo synthesis rather in brain microvessels and not in the CP.

## Figures and Tables

**Table 1:** The scheme of the experiment.

	Group	No. of animals	Experimental treatment I (iv.)	Dose (mg/animal)	Experimental treatment II (iv.)	Dose (ng/kg)
1	Control	6	NaCl	0	NaCl	0
2	LPS treated	6	NaCl	0	LPS	400
3	Donepezil treated	6	Donepezil	2.5	NaCl	0
4	Neostigmine treated	6	Neostigmine	0.5	NaCl	0
5	Donepezil + LPS treated	6	Donepezil	2.5	LPS	400
6	Neostigmine + LPS treated	6	Neostigmine	0.5	LPS	400
Total number of animals		36				

**Table 2:** Full names and abbreviations of all genes analysed by Real-Time PCR.

GenBank acc. no.	Gene	Amplicon size (bp)	Forward/reverse	Sequence 5′ → 3′	Reference
NM_001034034	*GAPDH* (glyceraldehyde-3-phosphate dehydrogenase)	134	Forward	AGAAGGCTGGGGCTCACT	[8]
			Reverse	GGCATTGCTGACAATCTTGA	
U39357	*ACTB* (beta actin)	168	Forward	CTTCCTTCCTGGGCATGG	[8]
			Reverse	GGGCAGTGATCTCTTTCTGC	
BC108088.1	*HDAC1* (histone deacetylase1)	115	Forward	CTGGGGACCTACGGGATATT	[8]
			Reverse	GACATGACCGGCTTGAAAAT	
X54796.1	*IL-1β* (interleukin 1-beta)	137	Forward	CAGCCGTGCAGTCAGTAAAA	[8]
			Reverse	GAAGCTCATGCAGAACACCA	
NM_001206735.1	*IL1R1* (interleukin 1 receptor, type I)	124	Forward	GGGAAGGGTCCACCTGTAAC	[8]
			Reverse	ACAATGCTTTCCCCAACGTA	
NM_001046210.1	*IL1R2* (interleukin 1 receptor, type II)	161	Forward	CGCCAGGCATACTCAGAAA	[28]
			Reverse	GAGAACGTGGCAGCTTCTTT	
NM_001308595.1	*IL1RN* (interleukin 1 receptor antagonist)	145	Forward	AGGATCTGGGATGTCAACCA	[28]
			Reverse	CATGGATCCCCAGGAACATA	
NM_001009392.1	*IL6* (interleukin 6)	165	Forward	GTTCAATCAGGCGATTTGCT	[8]
			Reverse	CCTGCGATCTTTTCCTTCAG	
NM_001110785	*IL6R* (interleukin 6 receptor)	149	Forward	TCAGCGACTCCGGAAACTAT	[8]
			Reverse	CCGAGGACTCCACTCACAAT	
XM_004016974	*IL6ST* (glycoprotein 130)	139	Forward	GGCTTGCCTCCTGAAAAACC	[29]
			Reverse	ACTTCTCTGTTGCCCACTCAG	
NM_001024860	*TNF* (tumor necrosis factor)	153	Forward	CAAATAACAAGCCGGTAGCC	[8]
			Reverse	AGATGAGGTAAAGCCCGTCA	
NM_174674	*TNFRSF1A* (tumor necrosis factor receptor, type 1)	137	Forward	AGGTGCCGGGATGAAATGTT	[8]
			Reverse	CAGAGGCTGCAGTTCAGACA	
NM_001040490	*TNFRSF1B* (tumor necrosis factor receptor, type 2)	122	Forward	ACCTTCTTCCTCCTCCCAAA	[8]
			Reverse	AGAAGCAGACCCAATGCTGT	
BC_149340	*CHRNA7* (neuronal acetylcholine receptor subunit alpha-7)	114	Forward	TGGAAGCCAGACATTCTCCT	[7]
			Reverse	GATGCCTGGAGGGAGGTACT	

**Table 3:** Effect of lipopolysaccharide (LPS; 400 ng/kg; iv.) and acetylcholinesterase inhibitors: donepezil (2.5 mg/animal; iv.) and neostigmine (0.5 mg/animal; iv.) injections on the relative gene expression (mean ± SEM; *n* = 6 animals per group) of proinflammatory cytokines, their corresponding receptors, and neuronal acetylcholine receptor in the preoptic area of the ovine hypothalamus.

Gene	Preoptic area of the hypothalamus					
	Control	Don.	Neo.	LPS	Don. + LPS	Neo. + LPS
*IL-1β*	1 ± 0.1^A^	1.2 ± 0.1^A^	0.9 ± 0.1^A^	4.4 ± 0.4^C^	3.2 ± 0.3^B^	3.1 ± 0.2^B^
*IL1R1*	1 ± 0.2^A^	1 ± 0.2^A^	1.1 ± 0.2^A^	1.7 ± 0.2^B^	1.4 ± 0.2^AB^	1.4 ± 0.1^AB^
*IL1R2*	1 ± 0.1^A^	0.8 ± 0.1^A^	1.1 ± 0.1^A^	1.6 ± 0.2^B^	1.8 ± 0.2^B^	1.5 ± 0.1^B^
*IL1RN*	1 ± 0.1^A^	0.9 ± 0.1^A^	0.9 ± 0.1^A^	2.7 ± 0.5^B^	2.1 ± 0.2^B^	2.3 ± 0.2^B^
*IL-6*	1 ± 0.1^A^	0.9 ± 0.1^A^	1.1 ± 0.2^A^	13.1 ± 0.1^C^	4.7 ± 0.6^B^	5.3 ± 0.3^B^
*IL6R*	1 ± 0.1^A^	0.8 ± 0.1^A^	0.9 ± 0.1^A^	1 ± 0.1^A^	1 ± 0.1^A^	0.9 ± 0.1^A^
*IL6ST*	1 ± 0.1^A^	0.9 ± 0.1^A^	1 ± 0.1^A^	1.3 ± 0.1^B^	1.3 ± 0.1^B^	1.1 ± 0.1^A^
*TNF*	1 ± 0.1^A^	1 ± 0.1^A^	1 ± 0.1^A^	1.4 ± 0.1^B^	1 ± 0.1^A^	1.1 ± 0.1^A^
*TNFRSF1A*	1 ± 0.1^A^	1 ± 0.2^A^	1 ± 0.2^A^	1.2 ± 0.1^A^	1.2 ± 0.1^A^	1.1 ± 0.1^A^
*TNFRSF1B*	1 ± 0.1^A^	1 ± 0.1^A^	1 ± 0.1^A^	1.4 ± 0.1^B^	1.2 ± 0.1^AB^	1.2 ± 0.1^AB^
*CHRNA7*	1 ± 0.1^A^	1 ± 0.1^A^	1 ± 0.1^A^	1.3 ± 0.1^B^	1.2 ± 0.1^AB^	1.2 ± 0.1^AB^

*IL-1β*: interleukin-1*β*; *IL1R1* and *IL1R2*: interleukin receptor types 1 and 2; *IL1RN*: interleukin-1 receptor antagonist; *IL-6*: interleukin-6; *IL6R*: glycoprotein 130; *TNF*: tumor necrosis factor; *TNFRSF1A* and *B*: tumor necrosis factor receptor types 1 and 2; *CHRNA7*: neuronal acetylcholine receptor subunit alpha-7. The gene expression data were normalised to the average relative level of gene expression in the control group of ewes, which was set to 1.0. Different capital letters indicate significant (*p* < 0.05) differences according to a two-way ANOVA followed by Fisher's post hoc test.

**Table 4:** Effect of lipopolysaccharide (LPS; 400 ng/kg; iv.) and acetylcholinesterase inhibitors: donepezil (2.5 mg/animal; iv.) and neostigmine (0.5 mg/animal; iv.) injections on the relative gene expression (mean ± SEM; *n* = 6 animals per group) of proinflammatory cytokines and their corresponding receptors in the ovine choroid plexus.

Gene	Choroid plexus					
	Control	Don.	Neo.	LPS	Don. + LPS	Neo. + LPS
*IL-1β*	1 ± 0.2^A^	0.6 ± 0.1^A^	0.5 ± 0.1^A^	4.3 ± 1.5^B^	4 ± 0.7^B^	5.7 ± 1.4^B^
*IL1R1*	1 ± 0.2^A^	0.8 ± 0.1^A^	0.9 ± 0.1^A^	2.1 ± 0.5^B^	2.2 ± 0.3^B^	2.1 ± 0.2^B^
*IL1R2*	1 ± 0.2^A^	0.6 ± 0.1^A^	1.1 ± 0.3^A^	2.9 ± 0.7^B^	3.7 ± 0.7^B^	4.4 ± 0.9^B^
*IL-1RA*	1 ± 0.1^A^	1 ± 0.1^A^	1.3 ± 0.1^A^	9.4 ± 0.6^B^	12.4 ± 1.3^B^	12.7 ± 2.5^B^
*IL-6*	1 ± 0.4^A^	0.9 ± 0.1^A^	1.2 ± 0.3^A^	84 ± 27^B^	84 ± 9^B^	123 ± 40^B^
*IL6R*	1 ± 0.1^AC^	1 ± 0.1^A^	1.1 ± 0.0^A^	0.8 ± 0.1^BC^	0.9 ± 0.1^ABC^	0.7 ± 0.1^B^
*IL6ST*	1 ± 0.2^A^	0.7 ± 0.1^A^	0.9 ± 0.1^A^	1.7 ± 0.2^B^	2 ± 0.2^B^	1.8 ± 0.4^B^
*TNF*	1 ± 0.1^A^	0.7 ± 0.1^A^	1 ± 0.1^A^	1.9 ± 0.6^B^	1.6 ± 0.2^B^	1.9 ± 06^B^
*TNFRSF1A*	1 ± 0.1^A^	0.8 ± 0.1^A^	1 ± 0.1^A^	1.3 ± 0.1^B^	1.4 ± 0.1^B^	1.4 ± 0.1^B^
*TNFRSF1B*	1 ± 0.1^AB^	0.8 ± 0.1^A^	0.8 ± 0.1^A^	1.6 ± 0.3^C^	1.7 ± 0.3^C^	1.4 ± 0.2^BC^

*IL-1β*: interleukin-1*β*; *IL1R1* and *IL1R2*: interleukin receptor types 1 and 2; *IL1RN*: interleukin-1 receptor antagonist; *IL-6*: interleukin-6; *IL6R*: glycoprotein 130; *TNF*: tumor necrosis factor; *TNFRSF1A* and *B*: tumor necrosis factor receptor types 1 and 2; *TLR4*: toll-like receptor 4. The gene expression data were normalised to the average relative level of gene expression in the control group of ewes, which was set to 1.0. Different capital letters indicate significant (*p* < 0.05) differences according to a two-way ANOVA followed by Fisher's post hoc test.

## Data Availability

The data used to support the findings of this study are available from the corresponding authors upon request.
